# Genetic attributes of Iranian cystic fibrosis patients: the diagnostic efficiency of CFTR mutations in over a decade

**DOI:** 10.3389/fgene.2023.1140034

**Published:** 2023-05-18

**Authors:** Amin Hosseini Nami, Mahboubeh Kabiri, Fatemeh Zafarghandi Motlagh, Tina Shirzadeh, Negar Fakhari, Ali Karimi, Hamideh Bagherian, Mojdeh Jamali, Shahrzad Younesikhah, Sara Shadman, Razie Zeinali, Sirous Zeinali

**Affiliations:** ^1^ Department of Biotechnology, College of Science, University of Tehran, Tehran, Iran; ^2^ Dr. Zeinali’s Medical Genetics Laboratory, Kawsar Human Genetics Research Center, Tehran, Iran; ^3^ Max Planck Institute for Brain Research, Frankfurt am Main, Germany; ^4^ Faculty of Life Sciences and Biotechnology, Shahid Beheshti University, Tehran, Iran

**Keywords:** autosomal recessive, CFTR mutation, cystic fibrosis, genetic diagnosis, newborn screening, population genetics

## Abstract

**Objectives:** Cystic fibrosis (CF) is the most prevalent autosomal recessive disorder among Caucasians. Mutations in the cystic fibrosis transmembrane conductance regulator (CFTR) gene cause this pathology. We, therefore, aimed to describe the CFTR mutations and their geographical distribution in Iran.

**Method:** The mutation spectrum for 87 families from all Iranian ethnicities was collected using ARMS PCR, Sanger sequencing, and MLPA.

**Results:** Mutations were identified in 95.8% of cases. This dataset revealed that the most frequent mutations in the Iranian population were F508del, c.1000C>T, c.1397C>G, c.1911delG, and c.1393-1G>A. In addition, we found weak evidence for Turkey being the possible geographical pathway for introducing CFTR mutations into Iran by mapping the frequency of CFTR mutations.

**Conclusion:** Our descriptive results will facilitate the genetic detection and prenatal diagnosis of cystic fibrosis within the Iranian population.

## 1 Introduction

Cystic fibrosis [CF (OMIM: #219700)] is a well-known life-limiting hereditary disease caused by deleterious mutations in the cystic fibrosis transmembrane conductance regulator (CFTR) gene on chromosome 7 (Ratjen et al., 2015). More than 72,000 cases of CF have been reported worldwide (Jackson and Goss, 2018), with the Caucasian population having the highest number of affected individuals (Cutting, 2005). CF has an uneven distribution internationally and amongst different ethnic groups. Interestingly, Japan has a very low incidence rate of about one in every 350,000 (Yamashiro et al., 1997). However, the prevalence of CF increases when analyzing Western populations. In Europe, the incidence spectrum is between 1:97,000 and 1:3,400 (Latvia and Ireland, respectively) (Farrell, 2008). The US also has a high incidence rate, with one in every 4,000 births (Scotet et al., 2020) and variability among ethnic groups. The Indian immigrant population of the United States has one CF patient in 40,000 (da Silva Filho et al., 2021), and one in every 17,000 African–Americans is affected with CF (Scotet et al., 2020). These statistics are mainly based on studies performed on the Western populations. There is a lack of epidemiological investigations in other populations affected by CF, such as Iran.

The Cystic Fibrosis Mutation Database has published more than 2000 CFTR gene mutations, of which 380 are verified as pathogenic (Harvey et al., 2022). Phenylalanine deletion at position 508 (F508del) is the most frequent within this database (Lopes-Pacheco, 2019). Mutation analysis has shown that approximately 60% of European CF patients have at least one F508del mutation. This mutation is more prevalent in southeastern and northern European countries than in their southern counterparts. The frequency of F508del varies from 5% in Georgia to 81.4% in Albania and Denmark (Castellani et al., 2008; European Cystic Fibrosis Society, 2017). As a point of comparison, the prevalence of other common mutations worldwide is only around 1%, and the prevalence of approximately 20 mutations is below 1%. Most CFTR mutation variants are limited geographically to small areas (Zietkiewicz et al., 2014).

Iran is in the Middle East with a population of ∼80 million and is composed of diverse ethnicities (Danaei et al., 2019; Alicata et al., 2020). Mutation analysis yields improved results since the rate of consanguinity marriage is high across the country. Studies on thalassemia and deafness (Mahdieh et al., 2010; Rezaee et al., 2012; Valaei et al., 2018) have described a gradient shift in mutation types and frequencies, moving from northwest to southeast. This suggests that the northwestern Iranian population is more similar to Turkish and East Europeans than those in the south and southeast.

Using recent advances in genetic tools to detect mutations, we have collected the spectrum of CFTR mutations in 87 Iranian families from 22 out of 31 provinces in Iran. We used autozygosity or homozygosity mapping to validate and support our findings. We found five mutations with the highest prevalence and weak evidence for a northwest-to-southeast decrease in the prevalence of the CFTR mutations. This suggests a possible European source of the mutations.

## 2 Materials and methods

### 2.1 Sample collection

Eighty-seven families were referred to the Medical Genetics Lab at the Kawsar Human Genetics Research Center for genetic counseling between 2008 and 2021. The main reasons for the visits were disease confirmation, prenatal diagnosis, or carrier detection. Patients covered seven Iranian ethnicities (Gilaki, Kurd, Lur, Persian, Tabari, Turk, and Turkeman), and their forebearers were determined based on self- or parents’ reports. CF was detected based on the Gibson–Cooke sweat test with a threshold value of ≥60 mmol/L and CF-associated symptoms, i.e., recurrent pneumonia, meconium ileus, greasy stools, and salty-tasting skin from birth (Farrell et al., 2017). The institutional review board of the Kawsar Human Genetics Research Center approved the ethical aspects of the study. All patients or their legal guardians signed an informed consent form to be included in the study. Peripheral blood samples were collected in anticoagulant ethylenediaminetetraacetic acid (EDTA) tubes, and DNA samples were extracted using either the salting out or the proteinase K method (Miller et al., 1988; Ghaheri et al., 2016).

### 2.2 Point mutation analysis

Initially, we used ARMS PCR to detect the presence of common mutations (i.e., c.1521_1523delCTT; c.3909C>G; c.350G>A; and c.3846G>A) in the CFTR gene (Newton et al., 1989). The samples lacking the aforementioned mutations were subjected to Sanger sequencing. The entire coding and associated flanking region (100–200 bp) were sequenced using a 3,130/xl Genetic Analyzer (ThermoFisher Scientific, Foster City, CA, United States, TF). Furthermore, mutation nomenclature complied with the Human Genome Variation Society’s guidelines (denDunnen and Antonarakis, 2000). Mutations’ novelty and pathogenicity were investigated using different sites, software, and databases. These were HGMD^1^, CFTR2^2^—clinical and functional translation of CFTR, cystic fibrosis mutation database^3^, CFTR France^4^, and literature review.

### 2.3 Multiple ligation-dependent probe amplification (MLPA) analysis for the detection of large rearrangements in the CFTR gene

In patients with none or only one pathogenic allele, we searched for possible deletion/duplications in the CFTR gene using multiple ligation-dependent probe amplification (MLPA, MRC Holland, Amsterdam, the Netherlands). The SALSA MLPA Probemix P091 CFTR was used to analyze DNA samples according to the manufacturer’s recommendation (Schouten et al., 2002). The amplification product was transferred to an ABI 3130/xl Genetic Analyzer (TF) for capillary electrophoresis. Data analysis was performed using Coffalyser (MRC Holland) or Gene Marker^®^ software v.1.6 (TF).

### 2.4 Statistical analysis

#### 2.4.1 CFTR mutation number distribution across provinces in Iran

The data contained the total number of CFTR mutations in patients across 22 provinces in Iran. Since we did not have any information about the number of mutations within nine other provinces (Yazd, Chaharmahal and Bakhtiari, Bushehr, Kohgiluyeh and Boyer-Ahmad, Ilam, Alborz, Semnan, South Khorasan, and Sistan), we used the coordinate of their capital (longitude and latitude) to interpolate the number of mutations using a Biharmonic spline interpolation method (MATLAB griddata function).

We used the Borders Package (www.mathworks.com/matlabcentral/fileexchange/50390-borders) written by Chad Greene on MATLAB central to plot the types and frequencies of mutations in Iranian borders. We then used the color intensity to represent the number and fraction of mutations within each province (Figure 4). All analyses was performed using MATLAB 2021a (MathWorks, United States).

#### 2.4.2 Correlation between the longitudinal location of an Iranian province and the number of CFTR mutations

We also examined the relationship between the distance of each province’s capital from Turkey’s central point with the coordinate (35° 14′26.67″, 38° 57′26.43″N) using the haversine formula (Śmietanka et al., 2015) and the total number of CFTR mutations within a province. The Spearman correlation coefficient between the log-transformed number of mutations and the spherical distance from the central point in Turkey is −0.33, and the *p*-value is 0.6 (Pearson’s correlation coefficient = −0.31 with a *p*-value = 0.08).

## 3 Results

**FIGURE 1:**
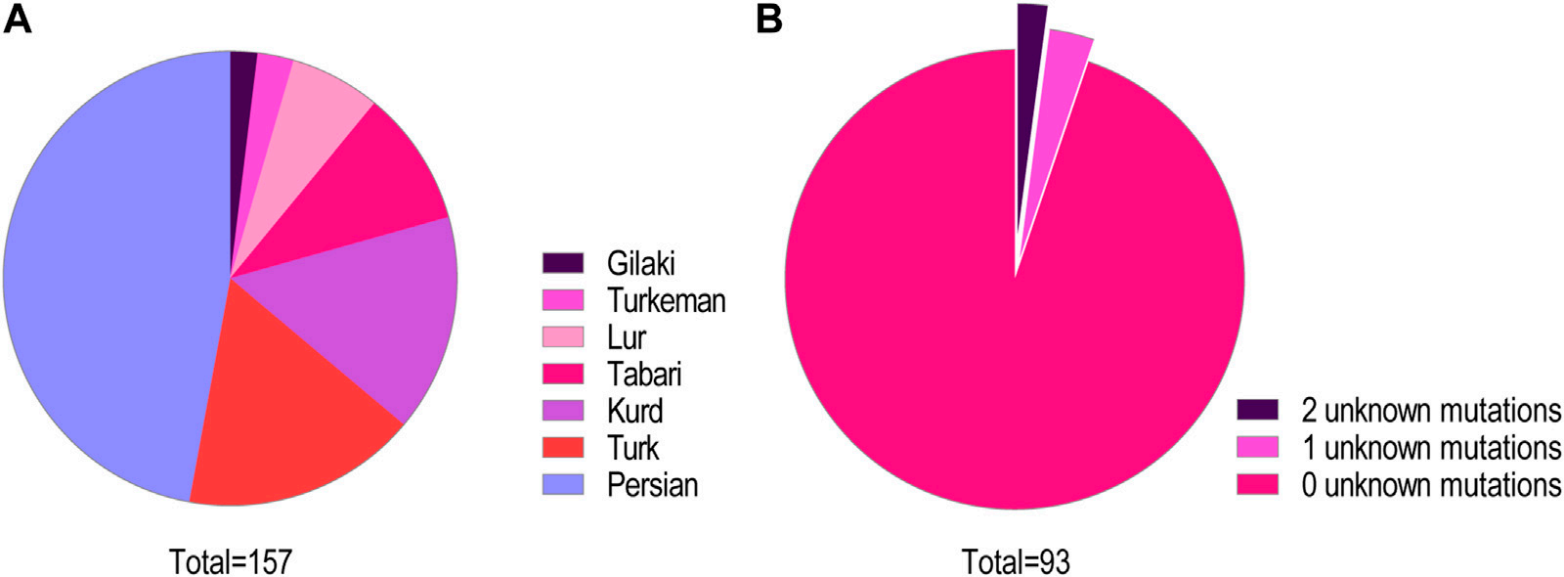
**(A)** Ethnicity distribution of CFTR mutations. The distribution of mutations in this cohort among the major ethnic groups in Iran is shown. **(B)** Classification of the study participants based on the number of detected pathogenic mutations. Among this cohort of patients and carriers, 88 cases (95%) had zero unknown mutations, three cases (3%) had one unknown mutant variant, and two cases (2%) remained with both mutations unknown.

**FIGURE 2:**
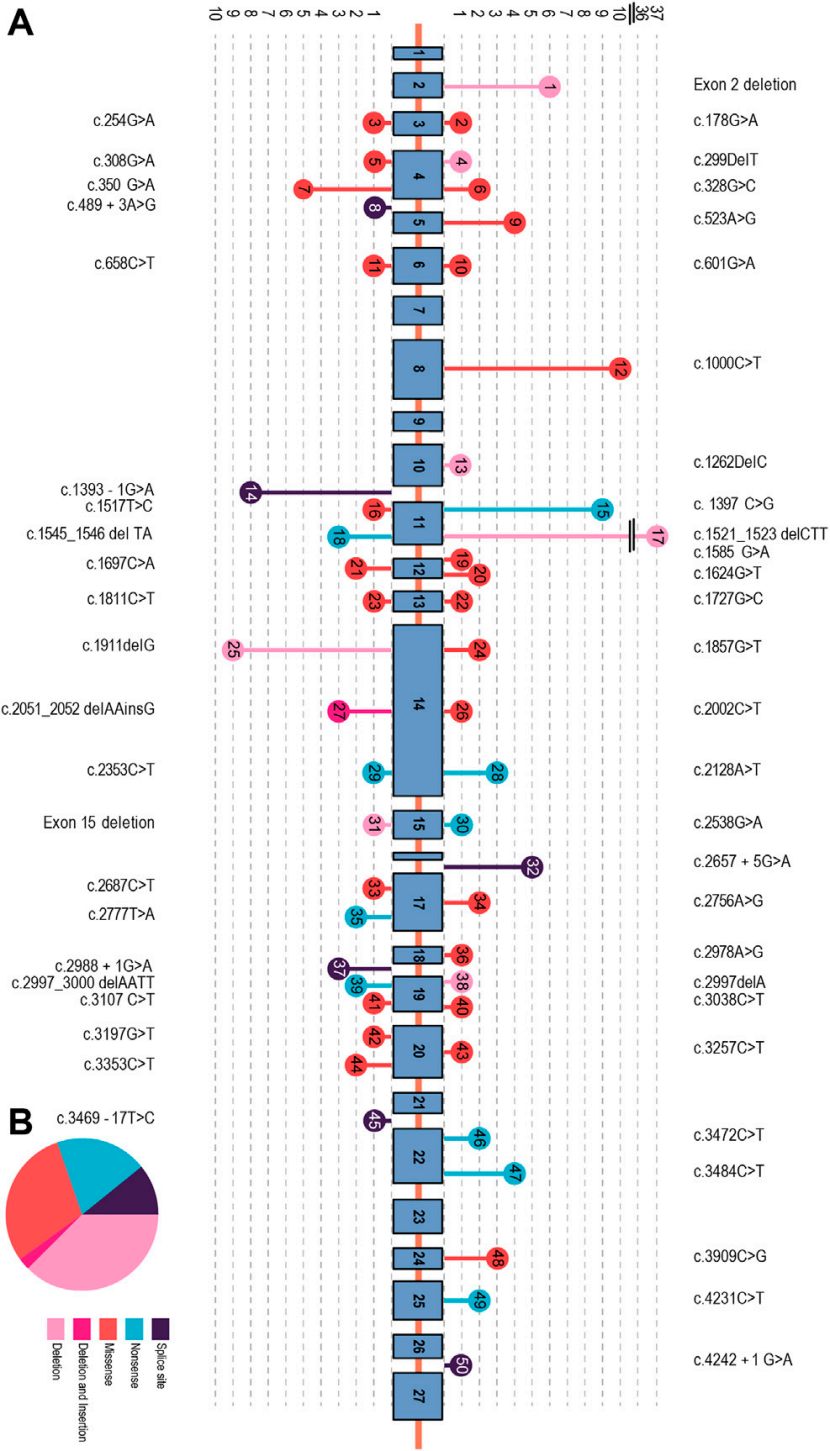
**(A)** Organization of the CFTR gene consisting of 27 exons, with the exon/intron position of each mutation. The circles in the arrowhead represent different mutations found in each exon/intron. Mutation types are indicated using different colors. **(B)** Mutation signature in the CFTR gene. The most prevalent point mutations were deletion (33%), missense (30%), non-sense (21%), and splicing (12%). Deletion–insertion (2%) mutations showed the lowest frequency in this cohort study.

We identified 93 individuals from 87 families with at least one type of mutation in the CFTR gene. These included 32 unrelated families. We identified 40 homozygous mutations among the participants and 26 compound heterozygous mutations. In two families, we described for the first time two novel mutations, one in a heterozygote form [i.e., c.299delT, (p.Leu100Profs*7)] and the other [i.e., c.1857G>T (p.Leu619Phe)] in a homozygotic form (Figure 2) (HosseiniNami et al., 2022). Three individuals had only one CFTR mutation despite their phenotypic consistency with CF. We were unable to identify the CFTR mutation in two individuals (Figure 1A). The unknown mutations most probably were deep intronic mutations since we had sequenced approximately 200 bases from either side of each exon. Subjects of this study were from seven different Iranian ethnicities (Figure 1B) covering 22 provinces of Iran.

Altogether, we characterized 157 CFTR mutant alleles out of 166 studied, which revealed 50 distinctive variations. The most prevalent mutations were deletions (33%), missense (30%), non-sense (21%), splicing (12%), and insertion/deletion (InDel, 2%) in this cohort study.

To understand the origin of the CFTR mutations, we analyzed the mutation frequency amongst CF patients in 22 provinces within Iran. In nine other provinces lacking data, we estimated the number of mutations using interpolation. The results from the box plot analysis indicated a maximum value of 15 and a minimum value of 2. The data distribution was skewed to the right, suggesting a greater concentration of values towards the lower end of the scale. Interestingly, we found weak evidence for a trend of decrease in the number of mutations moving from the northwestern to the southeastern provinces of Iran. Pearson’s and Spearman’s correlation coefficients between the distance of a province to the center of Turkey and its number of CFTR mutations were −0.31 (*p* = 0.08 for the null hypothesis of no correlation) and −0.33 (*p* = 0.06 for the null hypothesis of no correlation), respectively. Together with the previous studies showing similar patterns in different genes and mutations, this observation implies that the ancestral origin of mutations may have entered Iran through the neighboring northwestern regions (Figure 4).

## 4 Discussion

We aimed to collect the largest cohort of Iranian CF patients and carriers reported in the present study. The goal was to identify the most prevalent mutations within this sample of 87 families from 22 states and seven ethnic backgrounds. In addition, we found weak evidence for gradients in the geographic distribution of F508del mutation. Our study supplements epidemiological and genetic studies on CF. It also highlights the utility of haplotyping to aid our future design of accurate prenatal diagnosis in the Iranian population.

Studies of CFTR variants and their prevalence are firmly established in European and American populations, and these studies are limited in other regions, such as the Middle East. We aim to fill this gap by mapping the mutation spectrum of the Iranian population based on our analysis using ARMS PCR, Sanger sequencing, and MLPA. The study covers most Iranian ethnicities in 22/31 provinces of the country.

The following mutations with a frequency over 5% were found: c.1000C>T (6.33%), c.1397C>G (5.73%), c.1911delG (5.73%), and c.1393-1G>A (5.06%). However, we found the F508del to be the most frequent mutation in Iran, and it accounts for 23% of all the mutations. The observed frequency of F508del is higher than previously reported from Iran (Elahi et al., 2006; Alibakhshi et al., 2008). This is still below the levels observed in most of Western Europe (∼60%). It exceeds 80% in other countries, such as Albania (81.4%), Croatia (81.3%), and Denmark (81.4%) (European Cystic Fibrosis Society, 2017). Our findings show that 54% of delF508 alleles are observed in patients from the northwest and west of Iran, bordering Turkey. Turkey is the shortest route connecting Iran to the rest of Europe. This weak evidence for high regional density in the western provinces of Iran fits well with the source of the delF508 mutation being the European neighbors of Turkey. The data suggest a gradual frequency decline for this mutation in Iran from its western border to the east.

Previously, c.1000C>T (R334W) had been reported as a common mutation in Iran with 2.9% occurence (Morral et al., 1994); however, we found an increased frequency in our sample (6.33%). The geographical distribution of c.1000 C>T exhibits a stark difference between one area and the rest of the country (Figure 2). Lorestan Province in the west of Iran holds 50% of the c.1000 C>T mutations (Figure 2). Karimi et al. reported that the frequency of the c.1000 C>T mutation is as high as 40% in the CF patients from western Iran, whereas delF508 is observed only in 18% of their cases (Karimi et al., 2018). It is suggested that this mutation has multiple unrelated origins (Morral et al., 1994) and has been reported in distinct populations (Estivill et al., 1997; des Georges et al., 2004; Chevalier-Porst et al., 1994; Collazo et al., 2009; Lay-Son et al., 2011), with a considerable incidence in Southern Europe and Latin America (Pérez et al., 2007; Castellani et al., 2008). c.1000C>T is not a frequent mutation except in Brazilians (frequency of 2.64%) (Pereira et al., 2019) and ethnic Russians (0.69%) (Petrova et al., 2020). This makes this mutation a specific feature of Iranian CF patients.

**FIGURE 3:**
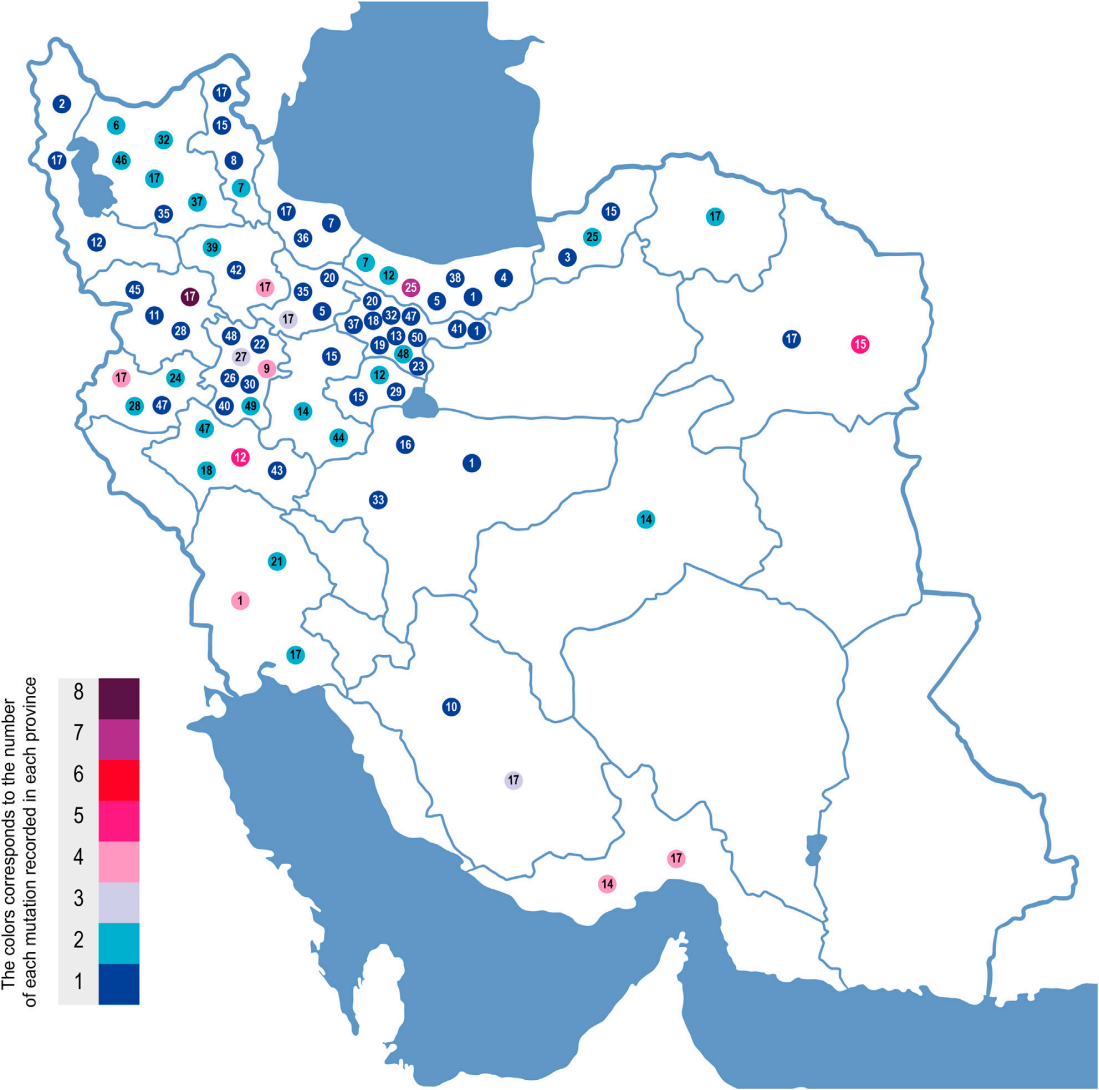
Geographic location of CFTR mutations in Iran based on CF patients genotyped in this study. On this map of Iran, the mutations are each indicated by a mutation number corresponding to the underlined mutation number in Figure 2. These numbers are placed in the geographic regions of the families where these mutations were identified. Each mutation’s color corresponds to the incidence number of that mutation in each province.

The third most frequent mutation in Iran is c.1397C>G (S466X), detected in 5.73% of the patients. This is similar to a previous study by Alibakhshi et al. reporting it to be 5.8% (Alibakhshi et al., 2008). Our study showed that 56% of this mutation is found in patients from Khorasan Razavi, a northeastern province of Iran (Figure 3). An earlier investigation of CFTR in the east of Iran reported it as the second most prevalent mutation in eastern Iran following the delF508 (MehdizadehHakkak et al., 2013). The c.1397C>G mutation is rare worldwide and more likely to be observed in Turkish, Greek, and Indian populations (Castellani et al., 2008). Moreover, 0.88% of Brazilians (Pereira et al., 2019), 0.86% in Serbians and Montenegrins (Radivojevic et al., 2004), 0.6% of African–Americans (Schrijver et al., 2016), and 0.72% of ethnic Russians (Lay-Son et al., 2011) also have this mutation.

Other relatively high-frequency mutations were c. 1911 del G and c. 1393-1 G>A. They were observed in 5.73% and 5.06% of the CFTR alleles, respectively. All c.1991delG (legacy name 2043delG)-mutated alleles were detected in northern Iran, and 78% were from Mazandaran Province with a Tabari origin. The prevalence of c.1991delG appears consistent with an earlier study of the CFTR mutation in the north (second most common mutation in that region (EsmaeiliDooki et al., 2015)). It is also a common mutation in Turkey (Onay et al., 2001), Saudi Arabia (Banjar et al., 2020), and Bahrain (31%). Eskandarani et al. proposed the possibility of the emergence of this mutation from Bahrain to the world, since most Bahraini CF patients have this mutation, with other regions only showing a low incidence rate (Eskandarani, 2002). This contrasts with our observation of the mutation, mainly in the north of Iran.

The earliest report on c.1393-1 G>A (legacy name 1525-1G > A) was in a patient of Indo-Iranian origin (Dörk et al., 1993). Based on our findings, 50% of the patients with this mutation are residents of Hormozgan in the south of Iran (Figure 3). This splice acceptor mutation is rare worldwide, with the main reports of Asian origin (Nikolic et al., 2014), such as Sri Lankan (Indika et al., 2019), Afghani (Ramalho et al., 2003), Pakistani (Wahab et al., 2004) (Miller et al., 1988), Indian (Ashavaid et al., 2005), and Palestinian populations (Siryani et al., 2015).

We found five additional mutations with a relative frequency from 2% to 5%. Among them, the deletion of exon 2 has the highest frequency. We observed this deletion in 3.8% of the mutated alleles. Another study of 527 Turkish patients showed that 63% also had an exon 2 deletion (Duz and OzyavuzCubuk, 2021). The other 40 mutations detected in our study have a frequency of less than 2%. Among them are c.1624G>T and c.3909C>G, with frequencies of 1.27% and 1.90%, respectively. Interestingly, these two mutations are among the top five most common mutations worldwide, with a frequency >1% (Bobadilla et al., 2002).

Three patients had clear indications of CF, with one undetected mutation using different techniques excluding intron sequencing. They only carried delF508, c.2777T>A, and c.3472C>T on one of their chromosomes. All three patients had positive sweat test results. Their other mutation should have been a deep intronic mutation beyond 200 base pairs from either side of each exon, since their MLPA results were normal. Another explanation could be the notion of the “manifesting carriers” phenomenon, when heterozygous CFTR mutation carriers may manifest CF-related symptoms (Miller et al., 2020). In addition, no mutation was found in two patients with positive sweat test results. The uncharacterized mutations might be located in the CFTR gene’s deep intronic regions or regulatory regions. However, we have analyzed an extended exonflanking region of 200 bp and utilized MLPA for negative point mutation results.

**FIGURE 4:**
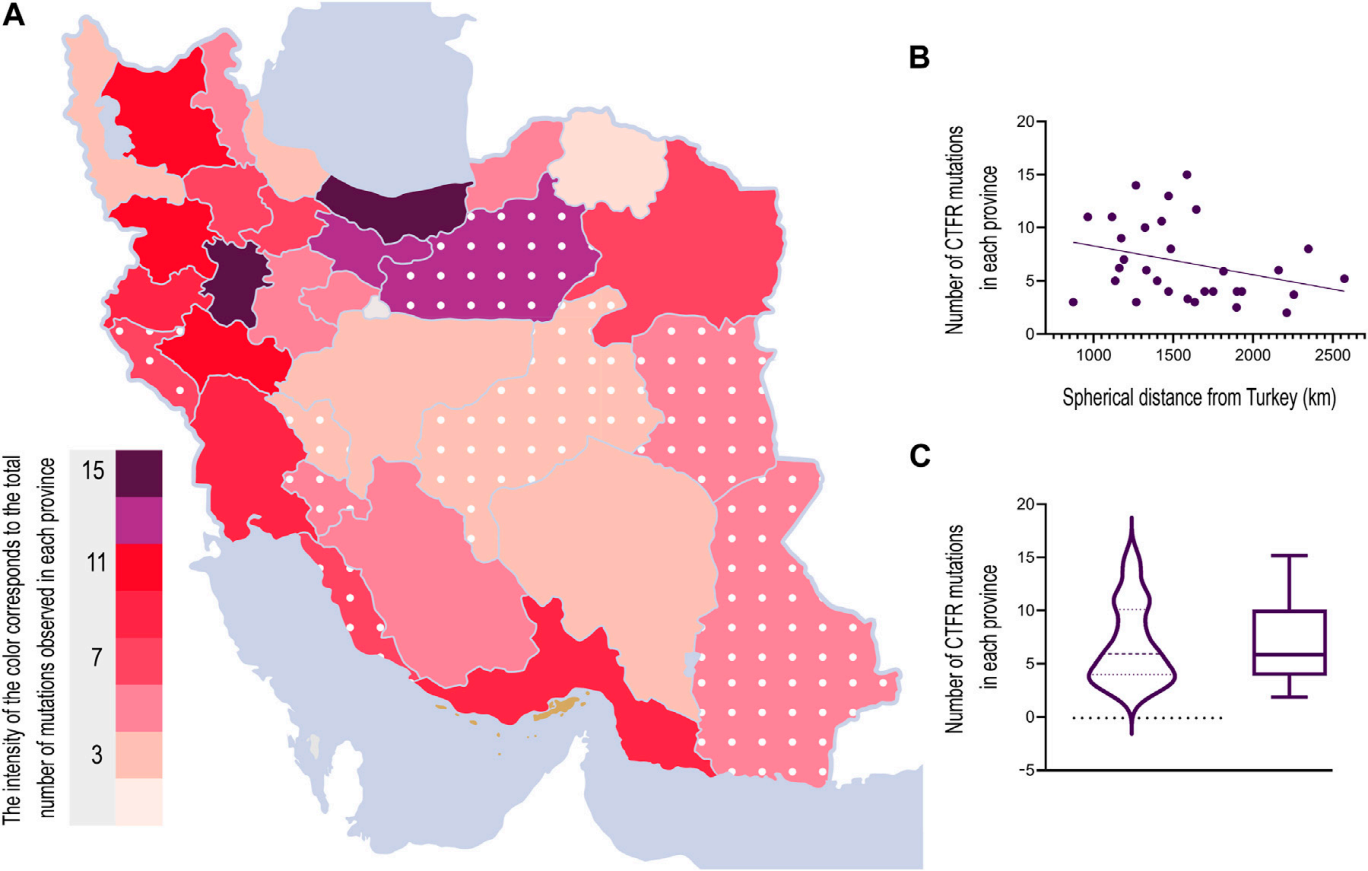
**(A)** Heat map of mutation number in each province of Iran. Different colors correspond to the number of mutations in each province. The interpolated mutation numbers for nine provinces are included and indicated with white dots. **(B)** Number of mutations’ correlation with the distance from Turkey indicates proximity to the northwest of Iran. Statistical significance (*p* < 0.1) was calculated using an unpaired, two‐tailed *t*-test. **(C)** Comparison between a box plot and a violin plot, displaying the data distribution with a minimum of 2 and a maximum of 15. The visualization suggests a right-skewed distribution, with a higher concentration of data toward the minimum value and a long tail toward the maximum value.

Most CFTR mutations are reported to have originated in Western countries (Castellani et al., 2008). This is implied in the distribution of the mutations found in the current study. There is a weakly significant decrease (significant at a threshold of 0.1) in the number of mutations as we move from the northwest to the southeast of Iran (Figure 4). However, the number of samples was limited within each province. This trend needs to be investigated further in future studies, with a higher number of patients analyzed in each province.

Iran makes up 1% of the world’s population (Mirzaei et al., 2020) and is the second most populated country in the Middle East, following Egypt (Danaei et al., 2019). It is located in a region on the Silk Road with different migrations and the influx of population as a result of various historical events, which highlights the valuable genomic information in the Iranian population (Fattahi et al., 2019). Several ethnicities with different dialects, languages, and religions form Iran’s present-day population. Recent studies indicate that the Iranian population is highly heterogeneous yet genetically distinctive (Mehrjoo et al., 2019). This explains the heterogeneity of CFTR mutations observed in the present study. Approximately 50 CFTR mutations account for nearly 96% of CFTR mutations in Iran. This notable mutation detection rate was a consequence of the use of sequencing and MLPA techniques accompanied by haplotyping and linkage analysis for a dataset covering over a decade. The previous mutation detection rate in Iranian patients was 53% (Elahi et al., 2006) and 81% (Alibakhshi et al., 2008) in studies based on populations of 60 and 69 individuals, respectively. We hope that this diverse characterization of the CFTR mutation in Iranian families will help genetic labs to provide and develop more accurate genetic counseling, prenatal diagnosis, and carrier detection for CF in the Iranian population worldwide.

## Data Availability

The original contributions presented in the study are included in the article, further inquiries can be directed to the corresponding author/s.

## Footnotes

1
www.hgmd.cf.ac.uk.

2
www.cftr2.org.

3
www.genet.sickkids.on.ca.

4
www.cftr.iurc.montp.inserm.fr/cftr.
